# Azithromycin use in labour to prevent sepsis among pregnant women undergoing vaginal delivery in Nigeria (AZIN-V): a study protocol for a hybrid type 2 effectiveness-implementation trial

**DOI:** 10.1136/bmjopen-2025-110719

**Published:** 2026-02-06

**Authors:** Bosede Bukola Afolabi, Christian Chigozie Makwe, Esther Oluwakemi Oluwole, Chisom Obi-Jeff, Eleanor J Mitchell, Aduragbemi Banke-Thomas, Titilope Adenike Adeyemo, Ajibola Ibraheem Abioye, Ejemai Amaize Eboreime, Abdulhadi Diyo Saidu, Udo Abali Okoro, Patricia Akintan, Chioma Stella Osuagwu, Chisom Florence Chieme, Teniola Lawanson, Anower Hossain, Kate Walker

**Affiliations:** 1 Department of Obstetrics and Gynaecology, University of Lagos College of Medicine, Lagos, Nigeria; 2 Department of Obstetrics and Gynaecology, Lagos University Teaching Hospital, Surulere, Nigeria; 3 Centre for Clinical Trials, Research and Implementation Science (CCTRIS), University of Lagos College of Medicine, Lagos, Nigeria; 4 Department of Community Health and Primary Care, University of Lagos College of Medicine, Lagos, Nigeria; 5 Department of Community Health and Primary Care, Lagos University Teaching Hospital, Surulere, Nigeria; 6 Brooks Insights, Abuja, Nigeria; 7 Department of Infectious Disease Epidemiology and International Health, Faculty of Epidemiology and Population Health, London School of Hygiene and Tropical Medicine, London, UK; 8 Nottingham Clinical Trials Unit, University of Nottingham School of Medicine, Nottingham, UK; 9 Maternal Adolescent Reproductive and Child Health Centre, Faculty of Infectious and Tropical Diseases, London School of Hygiene and Tropical Medicine, London, UK; 10 Department of Haematology and Blood Transfusion, University of Lagos College of Medicine, Lagos, Nigeria; 11 Department of Epidemiology, Harvard University T H Chan School of Public Health, Boston, Massachusetts, USA; 12 Department of Psychiatry, Faculty of Medicine, Dalhousie University, Halifax, Nova Scotia, Canada; 13 Federal Ministry of Health and Social Welfare, Abuja, Nigeria; 14 National Primary Healthcare Development Agency, Abuja, Nigeria; 15 Department of Paediatrics and Child Health, University of Lagos College of Medicine, Lagos, Nigeria; 16 Department of Paediatrics , Lagos University Teaching Hospital, Lagos, Nigeria; 17 Department of Medical Microbiology and Parasitology, University of Lagos College of Medicine, Lagos, Nigeria; 18 Department of Medical Microbiology and Parasitology, Lagos University Teaching Hospital, Surulere, Nigeria; 19 Warwick Clinical Trials Unit, University of Warwick, Coventry, UK; 20 University of Nottingham, Nottingham, UK; 21 UK Centre for Perinatal Research, School of Medicine, Nottingham University Hospitals NHS Trust—City Campus, Nottingham, UK

**Keywords:** Maternal medicine, Natural Childbirth, Sepsis, Antibiotics, Infection control, Implementation Science

## Abstract

**ABSTRACT:**

**Introduction:**

Nigeria has the highest number of maternal deaths globally, and maternal peripartum sepsis is one of the leading causes of maternal mortality. A single oral dose of azithromycin (AZM; 2 g) is safe and effectively reduces 33%–60% of maternal sepsis during planned vaginal birth in low- and middle-income countries (LMICs). However, the clinical and cost-effectiveness of oral AZM during vaginal birth in Nigeria remains unknown in the context of poor antimicrobial stewardship practices, significant antimicrobial resistance and healthcare financing. Evidence is also lacking on the standard care for the prevention of maternal sepsis among pregnant women undergoing vaginal births in Nigeria. The AZIN-V trial is a hybrid type 2 effectiveness-implementation trial to determine the safety, clinical and cost-effectiveness of intrapartum oral AZM versus usual care in the prevention of peripartum maternal sepsis. The trial will also examine the impact of implementation strategies in enhancing adherence to the oral AZM protocol during planned vaginal births and identify effective strategies to improve adherence (fidelity) to the protocol in real-world LMIC settings.

**Methods and analysis:**

This is a multicentre hybrid type 2 trial conducted in six Nigerian states: Ebonyi, Edo, Gombe, Kano, Kwara and Lagos. The study aims to simultaneously test the clinical and cost-effectiveness of AZM (clinical trial) and the impact of implementation strategies (implementation research) in Nigeria’s unique healthcare context. The clinical trial is a two-arm, cluster-randomised controlled trial conducted across 48 health facilities, randomly assigned (1:1) to either intrapartum administration of oral AZM (intervention group) or usual care—the current routine practice (control group). A total of 5040 study participants (2520 in each group) will be enrolled in the clinical trial. The implementation trial is a two-arm cluster non-randomised controlled trial conducted in 12 health facilities (1:1) allocated to either a bottom-up approach using the Plan-Do-Study-Act cycle or a usual top-down approach with a one-time training workshop and distribution of clinical guidelines, with both arms administering oral AZM during vaginal birth while assessing fidelity (primary outcome).

For the clinical trial, data will be analysed using intention-to-treat statistical methods. The cost-effectiveness outcome will be analysed using the Incremental Cost-Effectiveness Ratio. Implementation outcomes will be analysed using descriptive statistics and a thematic approach.

**Ethics and dissemination:**

This study has been approved by the National Health Research Ethics Committee, Nigeria (NHREC/01/01/2007-30/09/2024), the ethics committees of the participating health institutions (Lagos University Teaching Hospital Research Ethics Committee: ADM/DSCST/HREC/APP/6325; University of Ilorin Teaching Hospital Health Research Ethics Committee: ERC/PAN/2025/03/0581; University of Benin Teaching Hospital Health Research Ethics Committee: ADM/E22/A/VOL. VII/483117141; Aminu Kano Teaching Hospital Research Ethics Committee: AKTH/MAC/SUB/12 A/P-3/VI/2509 and Irrua Specialist Teaching Hospital Research Ethics Committee: ISTH/HREC/20241507/605), the Ministries of Health of the six states and the National Agency for Food and Drug Administration and Control. Written informed consent will be obtained from all eligible study participants before enrolment. Results will be shared with communities and policy stakeholders and through peer-reviewed journals and will be presented at conferences.

**Trial registration number:**

ISRCTN16415327.

**STRENGTHS AND LIMITATIONS OF THIS STUDY**

- This is a cluster-randomised clinical and cost-effectiveness trial of the intrapartum use of 2 g oral azithromycin (AZM) to prevent maternal sepsis among pregnant women undergoing vaginal birth in Nigeria, a country that accounts for 29% of all global maternal deaths.
- The trial will be conducted across six Nigerian states, one state selected from each of the country’s geopolitical zones and from different levels of healthcare delivery, which will ensure diversity and generalisability of the trial results.
- This trial represents a systematic evaluation of Plan-Do-Study-Act cycles for developing context-specific implementation strategies to enhance skilled health personnel’s adherence to intrapartum AZM protocols and could inform effective adoption and implementation in other comparable resource-constrained settings across Africa and beyond.
- As an open-label, cluster-design clinical trial, the participating sites were informed of their allocation, and this awareness may influence the clinical management practices or reporting behaviours across sites.
- The definition of usual care is not standardised across participating health facilities (study sites), and variations in routine practices for maternal sepsis prevention may introduce heterogeneity in the comparator arm.

## Introduction

 Maternal morbidity and mortality due to complications arising during pregnancy and childbirth remain a global health concern.^1^ Globally, an estimated 287 000 maternal deaths occurred in 2020, around 70% of which were in sub-Saharan Africa (SSA).^2^ Nigeria, the most populous country in SSA, bears the highest burden, accounting for nearly 29% of global maternal deaths,^2^ with peripartum sepsis as one of the leading causes.^3^,^6^ Maternal peripartum sepsis accounts for around 11% of all global maternal deaths, with a disproportionately higher burden in low- and middle-income countries (LMICs; 10%) compared with 5% in high-income countries.^1 7^ Maternal sepsis accounts for 17.3%–20.6% of maternal deaths in tertiary hospitals in Nigeria.^3 8^

To reduce maternal deaths from sepsis, the WHO recommends the use of antibiotics around birth to prevent and treat maternal peripartum infection in some clinical situations, including caesarean sections and operative vaginal births.^9 10^ Azithromycin (AZM), an inexpensive, heat-stable, orally administered broad-spectrum macrolide antibiotic with a prolonged half-life, is effective against Gram-positive and Gram-negative bacteria,^11^,^13^ responsible for maternal sepsis in SSA.^14^,^19^ While prophylactic use of antibiotics for caesarean birth is well-established and highly recommended,^20^,^22^ researchers continue to explore the clinical usefulness of intrapartum AZM in the prevention of maternal sepsis during vaginal birth.^23^,^25^ A single 2 g dose of AZM given orally is safe and cost-effective and has been shown to reduce maternal sepsis or death by 33%–60% during vaginal birth in LMICs, including SSA.^2324 26^,^28^

Although AZM has shown benefits in preventing maternal sepsis during vaginal births in other sub-Saharan African countries, several factors necessitate Nigerian-specific evidence before policy adoption. Nigeria’s distinctive antimicrobial resistance landscape, characterised by high resistance rates (up to 40% for Enterobacteriaceae and *Staphylococcus aureus*,^29 30^ the predominant causative organisms for maternal sepsis in SSA^31^) and high antibiotic exposure during pregnancy (due to antenatal infections and injudicious use of antibiotics), may reduce AZM’s effectiveness against most bacterial isolates responsible for maternal sepsis compared with previous trial settings.^32^,^34^ Additionally, Nigeria’s healthcare infrastructure varies considerably from primary health centres with limited laboratory capacity to tertiary hospitals with advanced diagnostic capabilities, potentially affecting both sepsis recognition and AZM’s clinical impact, while the economic implications of routine AZM use differ substantially in Nigeria’s healthcare financing context, where out-of-pocket payments constitute over 70% of health expenditures.^35^ A large randomised controlled trial is needed in Nigeria to assess the effectiveness and cost-effectiveness of a single dose of oral AZM during vaginal birth to reduce maternal sepsis and to guide clinical practice in Nigeria.

Beyond evidence on its effectiveness, there are also knowledge gaps regarding implementation. From an implementation perspective, the effective deployment of oral AZM for the prevention of maternal sepsis requires skilled health personnel’s (SHP’s) adherence to protocols in healthcare facilities.^36 37^ However, the typical top-down approach in Nigeria for guidelines or policy changes is to conduct a one-off training workshop and distribute the guidelines.^38^ This approach lacks evidence of sustained provider compliance, and no study has systematically evaluated implementation strategies to enhance SHP’s adherence to intrapartum AZM protocols in sub-Saharan settings. In addition, there is limited evidence on the facilitators, barriers and contextual factors influencing the implementation of oral AZM during vaginal birth to inform the design of context-specific implementation strategies and improve adherence.^23^ The Plan-Do-Study-Act (PDSA) cycle, a bottom-up approach, offers a promising strategy to develop, test and refine context-specific implementation strategies for the effective integration of evidence-based interventions, like intrapartum AZM prophylaxis, into routine practice in LMICs.^39^,^41^ Given the absence of AZM adoption in Nigeria for the prevention of maternal sepsis and uncertainty about effective implementation strategies,^42^ a systematic comparison of PDSA-generated strategies versus the usual strategy is needed to improve the fidelity of oral AZM in practice while upholding ethical standards and strategies to enhance SHP’s adherence across Nigeria’s health system and respecting the local medical context.

The AZIN-V trial is a hybrid type 2 effectiveness-implementation trial that simultaneously tests the clinical and cost-effectiveness of AZM while evaluating implementation. This study represents, to our knowledge, the first systematic evaluation of PDSA cycles for developing context-specific implementation strategies to enhance SHP’s adherence to intrapartum AZM protocols and would inform effective adoption and implementation in other comparable resource-constrained settings across Africa and beyond.

### Objectives

1. To assess the clinical effectiveness and safety of intrapartum administration of oral AZM compared with the current routine health-facility practice (usual care) for the prevention of maternal sepsis among pregnant women undergoing vaginal birth across multiple health facilities in Nigeria.
2. To estimate the incremental costs and cost-effectiveness of oral AZM compared with the current standard of care in preventing maternal sepsis among women undergoing vaginal birth across multiple health facilities in Nigeria.
3. To assess the effectiveness of SHP-tailored strategies to enhance the fidelity of oral AZM as part of a sepsis prevention protocol during vaginal birth across multiple health facilities in Nigeria.

## Methods and analysis

### Study design

This protocol is reported in line with the Standard Protocol Items: Recommendations for Interventional Trials 2025 reporting guidelines.^43^

**Figure 1:**
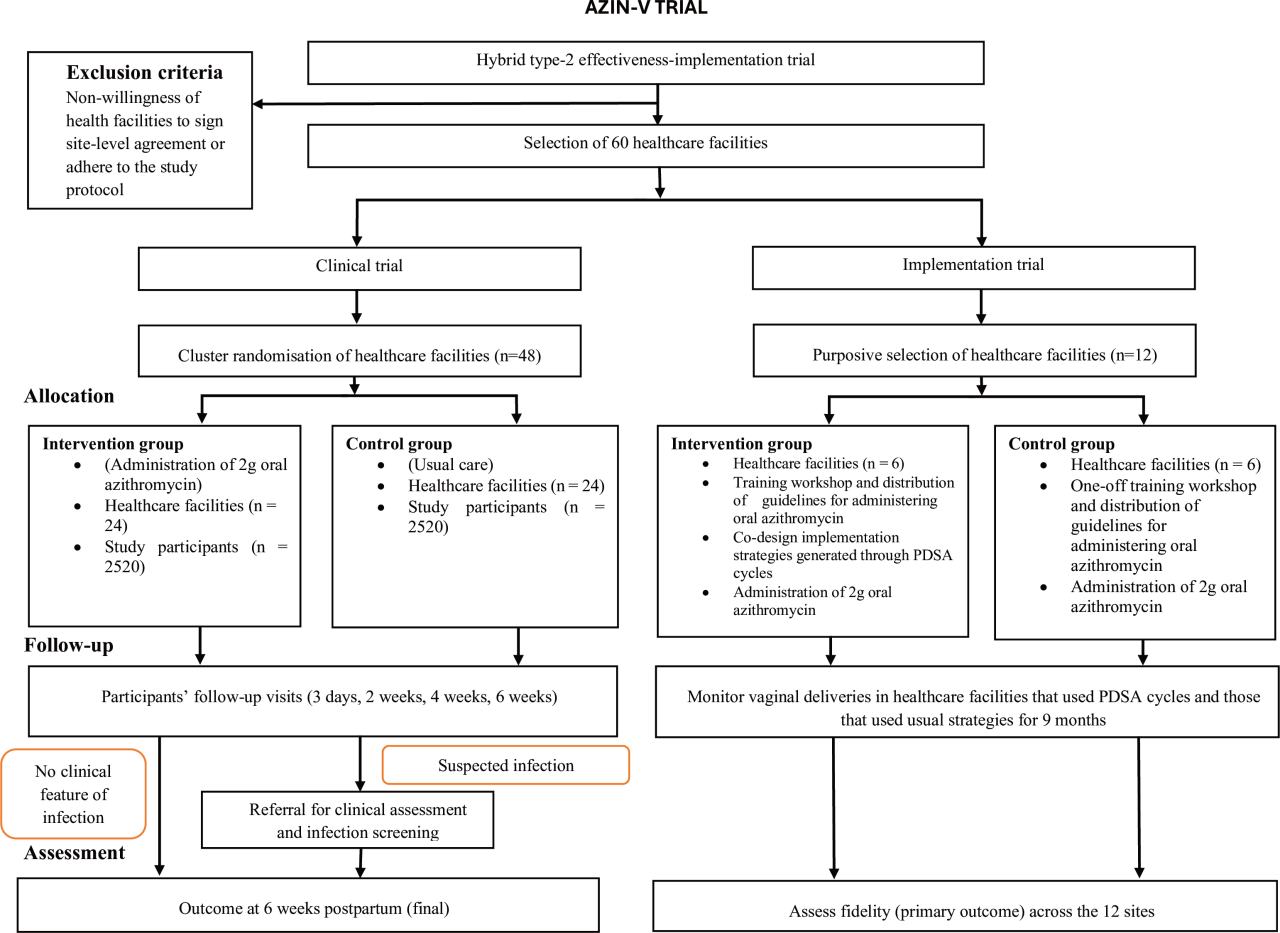
Trial flow diagram. PDSA, Plan-Do-Study-Act.

This is a hybrid type 2 effectiveness-implementation study.^44 45^ It comprises two components: (1) an intervention study to assess the effectiveness of oral AZM use in labour to prevent maternal sepsis among pregnant women undergoing vaginal birth and (2) implementation research to evaluate the implementation process (figure 1). AZIN-V includes two cluster-design parallel-group trials: a safety and effectiveness randomised trial conducted across 48 health facilities and an implementation trial conducted in 12 facilities.

### Study setting

The study is conducted across six Nigerian states, one state selected from each of the country’s geopolitical zones to ensure broad coverage: Ebonyi, Edo, Gombe, Kano, Kwara and Lagos. These states were selected based on differences in maternal healthcare indices and health system characteristics between the northern and southern regions. Study sites will include primary, secondary or tertiary health facilities that are either public or private. The list of study sites can be obtained from the trial registry (ISRCTN16415327).

### Sites

From the six states, 48 health facilities are included in the clinical trial. The implementation trial will include 12 health facilities from the six states. Sites will only participate in either the clinical or the implementation trial. Study sites are selected based on their state-level location to avoid serving the same geographic clusters or communities, thereby reducing the risk of cluster contamination. The selection process will ensure a within-state comparison across various levels of healthcare (primary, secondary and tertiary) and facility types: public (government-owned) and private.

### Clinical trial

#### Eligibility criteria

##### Sites

1. For sites to be eligible for either the clinical trial or implementation trial, they must have an antenatal clinic that serves at least 50 pregnant women per month and a labour ward that offers 24-hour vaginal birth services with an average of 20 vaginal births per month. Data were based on health facility records at potential study sites.
2. All selected study sites will sign a trial agreement before randomisation to commit to the assigned intervention group after randomisation. Thereafter, each study site is assigned a unique code for identification.

##### Participant inclusion criteria

1. Pregnant women with a live or stillborn singleton or twin pregnancy of at least 24+0 weeks’ gestation in the active phase of the first stage of labour (cervical dilatation of 4 cm or more) or the early (non-pushing) phase of the second stage of labour.
2. Pregnant women admitted in labour to the selected health facilities following spontaneous or induced labour with a planned vaginal birth.

##### Participant exclusion criteria

1. Pregnant women of gestational age <24 weeks.
2. Pregnant women who are not admitted to the facility with a plan to deliver vaginally.
3. Pregnant women in threatened preterm labour with no immediate plan for birth.
4. Pregnant women with a fever >38°C.
5. Pregnant women with a history of use of a macrolide antibiotic in the previous 3 days.
6. Pregnant women with a known allergy to AZM, its excipients or other macrolide antibiotics.
7. Pregnant women with a known history of cardiac disease (such as cardiac arrhythmia or cardiomyopathy).
8. Pregnant women with a planned caesarean birth.

### Intervention

For the clinical trial, a single 2 g oral dose of AZM will be administered to eligible participants in intervention sites. All other aspects of usual care in the health facility will remain the same (eg, handwashing, use of hand gloves and aseptic techniques). After obtaining written informed consent from an eligible participant, the health worker attending the birth will administer the oral AZM as a directly observed therapy.

### Control

Participants in control sites will receive usual routine care, as described above. AZM will not be provided to the control sites.

### Adherence and participant follow-up

After the potential participant has read the participant information sheet and consent form, but before she signs, the research staff will show her a sample study drug and confirm verbally that she is willing and able to take the AZM as prescribed (four 500 mg capsules) as part of the written informed process. Only if she is willing to commit to taking the pill and agrees to the scheduled follow-up visits will she be enrolled in the study after signing the consent form; otherwise, this is recorded as unwilling to give consent. Research nurses will witness the study participants taking the study drug. All participants (at intervention and control sites) will be followed up until discharge, with surveillance maintained (virtually and in person), and visits at 3 days, then at 2, 4 and 6 weeks after birth. Adherence will be monitored by the trial management group (TMG).

### Adverse drug event monitoring

Adverse events (AEs), including serious AEs (SAEs), are closely monitored and recorded on the electronic case report form throughout the duration of the trial, in accordance with Good Clinical Practice guidelines and all applicable regulatory requirements. At each study visit, participants are evaluated for AEs and asked about any symptoms that have occurred between study visits. Participants are advised and requested to report symptoms suggestive of AEs. An AE is defined as any unfavourable or unintended medical occurrence in any participant who has received the trial drug or usual care, regardless of whether it has a causal relationship with the intervention. SAE is defined as any event that (1) results in death; (2) is life-threatening; (3) requires hospitalisation or prolongation of existing hospitalisation; (4) leads to persistent or significant disability or incapacity or (5) constitutes a congenital anomaly/birth defect. AEs are documented according to the severity, duration, grade, relationship to trial drugs, interventions given and outcome. The costs of treating all adverse drug events related to trial drugs will be borne by the trial. Each AE/SAE is assessed by the site investigator for severity, expectedness and its relationship to the trial drug or study procedure (unrelated, unlikely related, possibly related, probably related or definitely related). All SAEs are reported to the Principal Investigator, who will, in turn, notify the trial sponsor and the Institutional Review Board/ethics committee in accordance with local regulatory requirements. Any serious, unexpected adverse drug reactions will also be reported promptly to the national drug regulatory authority in line with pharmacovigilance requirements. All study participants with AEs are managed and followed up until the resolution or stabilisation of the AE. Where necessary, additional medical information is obtained to ensure a comprehensive evaluation of the AE. Safety oversight during the trial is provided by a Data and Safety Monitoring Board (DSMB) and other independent safety monitors.

### Primary outcome

The primary clinical outcome is the incidence of maternal sepsis within 6 weeks (42 days) after birth.

Definition of primary clinical outcome: maternal sepsis is defined by the WHO as a life-threatening condition with organ dysfunction resulting from suspected or confirmed infection during pregnancy, childbirth, postabortion or the postpartum period. The WHO definition is used to define maternal sepsis as a suspected or confirmed infection based on table 1.

**Table 1:** Definition of maternal sepsis

Symptoms/signs	Definitions
Fever	>38°C
Hypothermia	<36°C
Plus, one or more signs of mild-to-moderate organ dysfunction, including:	
Tachycardia	≥110 beats per minute
Low blood pressure	<90/50 mm Hg
Tachypnoea	≥24 breaths per minute
Altered mental status	Altered consciousness, which may include coma, disorientation, etc.
Reduced urinary output	<500 mL/24 hours or<20 cc/hour (over several hours)

### Secondary clinical outcomes

1. Incidence of culture-confirmed maternal sepsis within 6 weeks of birth, defined as a positive blood culture in the presence of suspected maternal sepsis.
2. Incidence of specific maternal infection, defined as a positive microbiological culture from specific sites, including bacterial vaginosis, chorioamnionitis, endometritis, abdominal or pelvic abscess, mastitis or breast abscess, pneumonia or pyelonephritis (table 2).
3. Number of new prescriptions of antibiotics for a specific maternal infection after enrolment, for any reason, including bacterial vaginosis, chorioamnionitis, endometritis, abdominal or pelvic abscess, mastitis or breast abscess, pneumonia or pyelonephritis (table 2).
4. Number of new prescriptions of antibiotics for any reason (use of subsequent maternal antibiotic therapy after randomisation up to 6 weeks for any reason).
5. Incidence of neonatal sepsis within 28 days of birth, defined as proven or possible serious bacterial infection (PSBI) or pneumonia or meningitis. PSBI is determined using WHO criteria of severe chest in-drawing, fever, hypothermia, no movement at all or movement only on stimulation, feeding poorly or not feeding at all and/or convulsions.^46^ Clinical and laboratory signs of neonatal infection will also be considered for diagnosis, for example, eye infection, skin infection, omphalitis, urinary tract infection or respiratory rate ≥60 breaths per minute.
6. Incidence of stillbirth is defined as the birth of a baby with no signs of life at or after 24 weeks of gestation.
7. Incidence of neonatal death (death within 28 days of birth).
8. Duration of initial hospital stay for neonate, defined as the time from birth until the time of hospital discharge.
9. Duration of hospital stay for the mother, defined as the time from enrolment until discharge (in days).
10. Incidence of maternal readmission, or admission to a special care unit, for any other condition within 6 weeks of birth.
11. Incidence of neonatal readmission, or admission to a special care unit, for any other condition within 28 days of birth.
12. Incidence of adverse drug events (fainting or dizziness, nausea, vomiting, diarrhoea and dyspepsia) and other reported side effects.
13. Incidence of secondary postpartum haemorrhage, defined as excessive bleeding requiring intervention (such as surgical intervention or blood transfusion) from 24 hours after birth until 6 weeks post partum.

**Table 2:** Specified infections considered for maternal sepsis diagnosis

Type of infection	Description
Chorioamnionitis	Fever (>38°C) in addition to one or more of the following: fetal tachycardia≥160 beat s per minute, maternal tachycardia>100 beats per minute, tender uterus between contractions or purulent/foul-smelling discharge from the uterus prior to delivery.
Endometritis	Fever (>38°C) in addition to one or more of maternal tachycardia>100 beats per minute, tender uterine fundus or purulent/foul-smelling discharge from the uterus after delivery.
Wound infection	Purulent infection (superficial or deep infection, including necrotising fasciitis) of a perineal or caesarean wound with or without fever. In the absence of purulence, a wound infection requires the presence of fever (>38°C) and at least one of the following signs of local infection: pain or tenderness, swelling, heat or redness around the incision/laceration.
Abdominopelvic abscess	Evidence of pus in the abdomen or pelvis noted during open surgery, interventional aspiration or abscess imaging.
Pneumonia	Fever (>38°C) and clinical symptoms suggestive of lung infection, including cough and/or tachypnoea (>24 breaths per minute) or radiological confirmation.
Pyelonephritis	Fever (>38°C) and one or more of the following: urinalysis/dip suggestive of infection, costovertebral angle tenderness or confirmatory urine culture.
Mastitis/breast abscess	Fever (>38°C) and one or more of the following: breast pain; swelling; warmth; redness, abscess or infection or purulent drainage.

### Sample size

A sample size of 5040 (2520 in each group) is required to detect a 50% reduction in the maternal sepsis incidence rate from 6.5% to 3.25% due to AZM, at 90% power and a 5% level of significance. The sample size accounted for an intraclass correlation (ICC) of 0.011 and 20% loss-to-follow-up. The incidence rate of sepsis or severe systemic infection was found to be 6.5% in a study of maternal near-misses at the University of Port Harcourt, Rivers State, Nigeria.^47^ In a randomised clinical trial (RCT) of AZM versus placebo in Burkina Faso and The Gambia, the OR of maternal sepsis was 0.62 for any maternal sepsis and ~0.00 for bacterially confirmed sepsis.^25^ The incidence of sepsis in this study was much lower than in our setting (0.2% vs 6.5%). The ICC was derived from an analysis of the WHO Global Survey on Maternal and Perinatal Health.^48^ We require 48 health facilities and estimate each will enrol approximately 105 participants per facility within 10–12 months on average.

### Recruitment

All pregnant women will be approached during routine antenatal care (ANC) visits and on admission during labour by trained research staff who will provide study information and discuss participation. Study information will be provided and discussed using multiple formats, including videos, fliers, banners, participant information leaflets and eligibility assessment cue cards. To aid understanding, short information videos will be shown to potential participants during antenatal classes and/or after admission to the labour ward for childbirth. At the study sites, pregnant women admitted in the active phase of labour will be screened for eligibility to participate in the trial, and written consent will be obtained intrapartum from eligible participants before the onset of the pushing phase of labour. The informed consent form was translated into Hausa, Igbo, Yoruba and Pidgin English, the most popular languages used in the participating facilities.

### Randomisation

Sites were randomly assigned in a 1:1 ratio to either the intervention group or the control group. Randomisation was based on a minimisation algorithm that considered the state, facility level (primary, secondary and tertiary) and empirical postpartum use of antibiotics. Randomisation was undertaken by the statisticians, and the health facilities were informed of their random allocation before any participants were assessed for eligibility.

### Blinding

Due to the nature of the intervention and the cluster trial design, sites are not blinded to randomised allocation. The independent trial statistician will have access to blinded data until database lock to support the DSMB and the Trial Steering Committee (TSC).

### Data collection

Study participants are enrolled in labour and followed up with visits at 3 days, 2 weeks, 4 weeks and 6 weeks postpartum (table 3). To minimise missed visits and loss to follow-up, counselling sessions are extended to partners and family members when necessary and during follow-up visits. Reminders for follow-up appointments are sent via short message service and telephone. The details of participants’ home addresses are also collected at enrolment and updated during each visit. For participants who cannot be reached, community tracking will be done (a home-based monitoring system designed to locate participants in their homes). Consent for home visits and community tracking is obtained at enrolment.

**Table 3:** Schedule of activity

Study epoch	Antenatal	Labour	Postpartum				
Visit number	0	1a	1b	2	3	4	5
Time in study	ANC visits	During labour	After delivery, before discharge	3 days	2 weeks	4 weeks	6 weeks
Assessment window/study procedure			+12 hours	±1 day	±3 days	±5 days	±7 days
Awareness and sensitisation	X						
Counselling and provision of participant’s information leaflet	X	X					
Screening for eligibility		X					
Informed consent		X					
Assign study ID number		X					
Study intervention (AZM or usual care)		X					
Vital signs		X	X	X	X	X	X
Clinical lab evaluation*		X	X	X	X	X	X
Baseline information (mother) (sociodemographic, clinical, medical history, labour and delivery information, etc)		X	X				
Baseline newborn information (sex, birth weight, birth outcome, complications, etc)			X				
Maternal outcomes		X	X	X	X	X	X
Neonatal outcomes			X	X	X	X	X
AEs		X	X	X	X	X	X
Cost-effectiveness information		X	X	X	X	X	X
Participant EOS							X

Participants who booked for ANC will be counselled/consented during the antenatal period and consented/reconsented in labour.

* Clinical lab evaluation includes full blood count; electrolyte, urea and creatinine; procalcitonin; malaria parasite and microbiology culture as indicated.

AE, adverse event; ANC, antenatal care; AZM, azithromycin; EOS, end of study.

REDCap (Research Electronic Data Capture V.13.8.3, Vanderbilt University, Nashville, Tennessee, USA) is used to collect all relevant study data. Designated investigators and study staff will enter trial data using password-protected electronic devices. Outcome data are collected during labour and childbirth and up to 42 days postpartum. Baseline sociodemographic data are also collected.

In the case of suspected maternal and/or neonatal infection, participants are invited to the facility for detailed assessment and sample collection by the clinician, with relevant biological specimens collected: peripheral blood for blood culture, full blood count, blood film, malaria parasite, electrolytes, urea and creatinine, liver function tests and procalcitonin; midstream urine for microscopy, culture and sensitivity (MCS) and wound (perineal or caesarean) swab, drained abscesses swab, stool, sputum or tissue samples for MCS as appropriate. Microbiological samples are collected aseptically from the mother and newborn at multiple follow-up points, as required. These samples are cultured to identify bacterial species.

Maternal antibiotic usage data are also recorded, covering study and non-study antibiotics administered before birth, during the hospital stay and after discharge, up to 42 days postpartum to capture the full scope of antimicrobial exposure. Any AEs related to the intervention or pregnancy and childbirth are documented to monitor safety. All clinical and laboratory data are securely managed via an electronic data capture system (REDCap) with centralised quality control measures to ensure accuracy and consistency.

### Statistical methods and analysis

All analyses will be intention-to-treat unless specified. Categorical variables will be summarised as frequencies and percentages. Continuous variables will be presented with N (number of observations), means (SD) and/or medians (IQRs), depending on whether they are normally distributed. The primary outcome will be analysed using a random effects logistic regression model to estimate the treatment effect with 95% CI. The model will be adjusted for the stratification variables, along with any imbalance in key baseline variables. The secondary outcomes will be analysed using random-effects models depending on the distribution of the outcome. Missing data will be described explicitly with the number reported for each variable, and where appropriate, multiple imputation methods may be employed. An interim analysis will be conducted at the halfway point through the trial (50% of the target sample with the primary outcome). If the p value indicates an overwhelming effect (as evidenced by a p value less than 0.001—the Haybittle-Peto rule),^49^ the DSMB may request unblinding, and a recommendation to stop the trial may be considered.

### Cost-effectiveness analysis

An Incremental Cost-Effectiveness Ratio (ICER)—the additional cost per unit of health gain with AZM compared with usual care for individual participants as a component of the sepsis prevention protocol in healthcare facilities—is included.

Cost data related to birth and associated healthcare services are collected from both participants and health facilities involved in the study. From participants, cost data are collected after birth through structured interviews conducted before discharge and during any postpartum visits, up to 42 days. The data will include out-of-pocket expenses for birth consultations, medications, laboratory tests, hospital stays, transportation and any other costs incurred due to maternal or neonatal care, especially those related to AEs or complications. From the 48 health facilities of this clinical trial, cost data are collected through interviews and records review, involving facility managers, healthcare staff, administrators, laboratory scientists, pharmacists and other relevant personnel. This will cover direct and indirect costs associated with delivering care, including staff time, consumables, medications, laboratory and diagnostic services, infrastructure uses and overhead costs. Data on service utilisation, resource allocation and administrative expenditures relevant to maternal and neonatal care during the study period are extracted from facility records. This dual approach will enable a comprehensive estimation of the cost-effectiveness of the intervention from both patient and provider perspectives.

We will conduct a cost-effectiveness analysis to compare the costs and health benefits associated with AZM intervention compared with the usual care in reducing maternal sepsis among pregnant women undergoing vaginal birth from both patient and health provider perspectives. Cost elements for both arms will be comparatively assessed at an individual participant level, using a decision tree. Comparative analyses of the direct medical costs of the intervention and usual care, including the costs of administering a full course of treatment with both products (medicines, consumables and supplies), will be included. Analysis of all costs borne by participants from potential AEs and any loss of productivity while incapacitated by these AEs will be conducted for the study population (all enrolled women in the clinical trial). The ICER will be obtained from the mean values of costs and effectiveness. The CI for the ICER will be derived using non-parametric bootstrapping to quantify sampling uncertainty. Incremental costs and utilities in disability-adjusted life years averted will be determined as part of a cost-utility analysis. Uncertainty will be addressed by a univariate sensitivity analysis. In addition, a probabilistic sensitivity analysis, in which all parameters with uncertainty will be varied within their CIs, will be used to establish a credible interval for the estimated result.

### Implementation trial

#### Study design

The implementation trial is conducted as a two-arm, cluster non-randomised controlled trial with a parallel process evaluation. For this, 12 health facilities (clusters) were allocated to the intervention (PDSA cycle) and comparison (usual strategy) sites (figure 1), based on logistics and interest in facility-level outcomes (SHP’s adherence to oral AZM administration according to protocol).^50^ The six intervention sites will use the PDSA cycles to co-design, refine and adapt context-specific implementation strategies to enhance SHP’s adherence to oral AZM as part of a sepsis prevention protocol for maternal sepsis during vaginal birth. The six comparison sites will implement oral AZM during vaginal birth using the usual strategy for adopting new treatments—a one-off training workshop and guideline distribution. Then, fidelity in healthcare facilities that used PDSA cycles to generate implementation strategies will be compared with those that used the usual strategy to understand which strategies work best and how they are effective in various settings.

#### Theoretical framework

The implementation trial is guided by two frameworks: the Consolidated Framework for Implementation Research (CFIR) and the Reach, Effectiveness, Adoption, Implementation and Maintenance (RE-AIM). CFIR, a determinant framework comprising 39 constructs across five domains (Innovation, Outer Setting, Inner Setting, Individuals and Implementation Process), was chosen to understand barriers and facilitators influencing the implementation of oral AZM and the implementation outcomes.^4651^,^53^ On the other hand, the RE-AIM was selected as the evaluation framework to assess individual-level (reach and effectiveness) and setting-level (adoption, fidelity and sustainability) outcomes.^54 55^

#### Study sites

Our trial is implemented in 12 purposively selected sites within six states from a pool of facilities where a pre-site readiness assessment was conducted to account for health systems-based organisation factors. The inclusion criteria were (1) facility level (primary, secondary and tertiary) and facility ownership: public (government-owned) and private (individual, charity, faith-based or non-government organisation-owned); (2) provision of maternity services (ANC, childbirth and postnatal care services); (3) mixture of high, medium and low number of vaginal births and (4) location (rural, semiurban and urban).

#### Study population

Our study population consists of (1) SHP (eg, midwives, nurses, obstetricians) providing care during vaginal birth and (2) pregnant women (see *Participant inclusion and exclusion criteria*) giving birth vaginally across the 12 sites.

#### Sample size

Given that our implementation study uses PDSA as an implementation strategy to (1) facilitate fidelity to AZM according to the protocol and (2) generate context-specific learnings to improve quality of care—not for generalisation—the conventional approach to calculating sample size is not required.^56^ Instead, we will monitor all vaginal births in the 12 facilities for 9 months to assess fidelity.

#### Blinding

Blinding SHP within the selected health facilities is not feasible because they are directly involved in implementing the strategies to improve fidelity.^57^

#### Intervention: deployment of PDSA cycles to generate context-specific implementation strategies

At the PDSA sites, we will establish a multidisciplinary team (MDT) comprising a facility manager or head of department/unit, SHP, pharmacists, data managers and a patient representative. These MDTs will co-design, test and adapt implementation strategies generated through PDSA cycles to improve adherence to the oral AZM protocol for vaginal births. We will implement the PDSA cycle for 12 months, which involves four 1-day quarterly learning sessions and three alternating action periods using facility-generated data. During the learning sessions, MDTs will co-design implementation strategies using facility-generated data, suggest indicators to assess their performance and develop testing plans while reviewing progress and highlighting best practices. The action periods allow for the implementation, iterative testing and refinement of the co-designed strategies while monitoring and assessing progress towards adoption based on lessons learnt from previous testing. Technical support is provided to the MDTs throughout, and the process is documented using a PDSA documentation template. The co-designed implementation strategies are tested and refined for 9 months (ie, the three action periods) within the intervention period.

#### Comparison: usual strategy

At the non-PDSA sites, SHP in the selected health facilities will implement oral AZM during vaginal births using the usual strategy, which typically includes a one-off training workshop and distributing guidelines for administering the new treatment.

#### Outcome measures

The primary outcome is fidelity, with secondary outcomes as adoption, reach, feasibility, acceptability and sustainability (table 4). Fidelity (ie, SHP’s adherence to oral AZM according to protocol) is operationalised as the proportion of vaginal births in which oral AZM was administered according to the protocol during vaginal birth, using data extracted from facility records and observations to ensure accuracy. Adherence to oral AZM per protocol is defined as having ≥70% of the essential components, based on evidence of <70% non-compliance with antepartum and intrapartum practice guidelines in Nigeria^58^ and other countries and disciplines.^59 60^

**Table 4:** Implementation trial outcome, measure, data source and timeline

Outcomes	Measure	Data source	Timeline
Fidelity	The proportion of planned vaginal births in which oral AZM was administered according to the protocol/guideline	Health facility records and observation checklist	Midline and endline
	Factors influencing adherence to oral AZM administration according to the protocol	IDI with SHP	Endline
Reach	The proportion of eligible women who received oral AZM in planned vaginal delivery	Health facility records	Midline and endline
Adoption	SHP’s uptake of oral AZM in planned vaginal delivery as part of a sepsis prevention protocol	ORIC survey and KII with SHP and health facility managers	Baseline
	Factors that facilitated the adoption of oral AZM administration according to the protocol	IDI with SHP and health facility managers	Endline
Feasibility	The extent to which the implementation of oral AZM during planned vaginal delivery as part of a sepsis prevention protocol is considered feasible, given health facilities’ different needs and the availability and adequacy of required resources	KII with SHP and health facility managers	Baseline
		IDI with SHP and health facility managers	Endline
		Acceptability and feasibility of intervention measures, and FGD with MDT	Learning sessions 1 and 4
Acceptability	SHPs’ and women’s perception regarding administering and using oral AZM during planned vaginal delivery as part of a sepsis prevention protocol as agreeable, palatable or satisfactory	KII with SHP and health facility managers	Baseline
		IDI with SHP and women	Endline
Sustainability	The extent to which oral AZM during planned vaginal delivery becomes institutionalised as part of a sepsis prevention protocol	KII with health facility managers and SHP	Endline

AZM, azithromycin; FGD, focus group discussion; IDI, in-depth interview; KII, key informant interview; MDT, multidisciplinary team; ORIC, Organisational Readiness for Implementing Change; SHP, skilled health personnel.

#### Data collection methods

We will use quantitative and qualitative methods to assess the primary and secondary outcomes. Before the trial, we will assess the readiness of 12 healthcare facilities to adopt oral AZM during vaginal birth using the Organisational Readiness for Implementing Change (ORIC) validated survey.^61^ Using a semistructured interview guide, we will conduct key informant interviews to understand the barriers, facilitators and contextual factors influencing the implementation of oral AZM during vaginal birth in Nigerian health facilities guided by CFIR, as well as its feasibility and acceptability. We will also collect relevant contextual data, such as organisational characteristics, policy documents and healthcare system factors, to support exploring contextual factors. At the end of the PDSA cycles 1 and 4, we will use the validated 4-item scale: the Acceptability of Intervention Measure (AIM), the Feasibility of Intervention Measure (FIM)^62^ and focus group discussions using an open-ended semistructured discussion guide to collect data among the MDTs on the feasibility and acceptability of the co-designed implementation strategies. Data on fidelity are collected using facility records and an observation checklist deployed on REDCap. Additionally, we will conduct in-depth interviews (IDIs) at facilities with varying levels of adherence to the protocol to better understand the factors affecting fidelity and inform future improvements. Facility records will also be used to collect data on reach. Clinical data will not be collected from the implementation study sites.

#### Data analyses

The ORIC scores will be analysed descriptively. AIM and FIM will be summed with means calculated for each measure. Higher scores will indicate better acceptability and feasibility.^62^ Fidelity scores will be analysed using a linear mixed-effects model to compare the effectiveness of the two implementation strategies on fidelity. Subgroup analysis will also be conducted to provide additional insights into the effectiveness of the implementation strategy on fidelity in specific contexts. All quantitative data will be analysed using STATA V.18 (StataCorp LLC, College Station, Texas, USA). The qualitative interviews on facilitators, barriers and contextual factors to adopt oral AZM will be analysed using deductive methods based on CFIR domains and inductive methods using Braun and Clarke’s thematic analysis approach.^63^ Other qualitative interviews to assess fidelity, feasibility, acceptability and sustainability will also be analysed thematically using Braun and Clarke’s approach.^63^ Dedoose V.9.0.17 (SocioCultural Research Consultants, LLC, Los Angeles, California, USA) will support our qualitative analysis.

#### Integration

Quantitative and qualitative data will be integrated during data collection and analysis and merged during the interpretation phase to provide a comprehensive understanding of the effect of implementation strategies on fidelity and factors influencing implementation outcomes.

### Ancillary and post-trial care

All participants are covered by clinical trial insurance in case of serious adverse drug events arising from the use of trial drugs. There are no specific plans for post-trial care.

### Patient and public involvement

Before the trial commenced, IDIs were conducted at two health facilities, involving purposively sampled 10 healthcare personnel (five nurses, four doctors and one laboratory technician) and 20 pregnant women, to understand their views, expectations, concerns and suggestions regarding the AZIN-V trial. The trial protocol was adapted based on their feedback.

### Research governance

The TMG is responsible for ensuring high-quality delivery of the trial in accordance with the protocol and addressing problems promptly. The TMG includes the chief investigator, coinvestigators, trial manager and data manager. The TMG reports to the independent TSC and the independent DSMB. The TSC will provide independent oversight and advice to the trial, while the DSMB is responsible for monitoring the trial data, including safety and data integrity. The independent DSMB will monitor all SAEs and review SAE events by masked treatment group at each meeting and report to the TSC. The DSMB will consist of a global health physician (Christopher Robert Sudfeld, Harvard T.H. Chan School of Public Health, USA), a paediatrician (Martin Meremikwu, University of Calabar, Nigeria) and a medical biostatistician (Joanna Sturgess, London School of Hygiene and Tropical Medicine, UK). Auditing of research records will be conducted by the monitoring committees as outlined above, while financial auditing will be conducted by the Research Management Office and the Audit Department of the University of Lagos.

### Ethics and dissemination

#### Ethical considerations

Ethical approval has been obtained from the National Health Research Ethics Committee (NHREC), Nigeria (NHREC/01/01/2007-30/09/2024), the ethics committees of the participating health institutions (Lagos University Teaching Hospital Research Ethics Committee: ADM/DSCST/HREC/APP/6325; University of Ilorin Teaching Hospital Health Research Ethics Committee: ERC/PAN/2025/03/0581; University of Benin Teaching Hospital Health Research Ethics Committee: ADM/E22/A/VOL. VII/483117141; Aminu Kano Teaching Hospital Research Ethics Committee: AKTH/MAC/SUB/12 A/P-3/VI/2509; Irrua Specialist Teaching Hospital Research Ethics Committee: ISTH/HREC/20241507/605) and the states’ health ethics boards. Regulatory approval was obtained from the National Agency for Food and Drug Administration and Control. The trial is duly registered in the International Standard Randomised Controlled Trial Number (ISRCTN16415327) registry.

Written consent for participation, including consent for community tracking and assessing health records, is obtained from all eligible women (online supplemental material).^64 65^ Women aged 15–18 years are considered capable of providing consent, as they are considered emancipated for the purposes of this study, according to the guidelines of the NHREC and the 2016 WHO International Ethical Guidelines for Health-Related Research Involving Humans. Additional consent for de-identified data and samples is obtained for future studies or data sharing with secondary researchers.

This research protocol acknowledges the essential clinical responsibilities of health facility staff involved in the study, particularly in data collection and administration of the investigational drug to enrolled participants. To minimise disruption to their routine clinical duties, the following measures were implemented: (1) two trained research nurses are assigned per facility to assist with research tasks (data collection and participant tracking/follow-up) to ease the workload of regular clinical staff; (2) research activities are scheduled in coordination with facility management to avoid peak clinical hours and ensure uninterrupted patient care; (3) facility clinical staff’s participation in research is limited, clearly communicated and will not extend beyond their agreed working hours; (4) continuous monitoring is carried out to promptly identify and address any negative impact of research activities on clinical workflow and patient care and (5) participation of clinical staff in research tasks is voluntary, with the option to withdraw at any time without negative consequences.

Confidentiality of data obtained from participants will be maintained throughout the trial. All data collected at the study sites on REDCap are stored centrally in a password-protected electronic database by the senior data manager, who is the only person with access to the blinded data of all participants, collated centrally. Any approved modification to the protocol will be communicated to the investigators, and retraining of study staff on the amended protocol will be done as necessary.

#### Dissemination

The findings of this research will be shared through presentations at various scientific conferences in the fields of obstetrics, infectious diseases and public health, with publication in peer-reviewed journals and engagement with healthcare providers and relevant policymakers globally. Community engagement events and press releases will be used to communicate the results to the public and study participants.

### Trial status

The current protocol is V.3.0, 02 December 2025. The first participant was enrolled on 17 March 2025, and recruitment is projected to end by March 2026. The trial registration dataset is available on: https://www.isrctn.com/ISRCTN16415327.

## Supplementary material

10.1136/bmjopen-2025-110719 online supplemental file 1
