# Supplementary material for: Azithromycin use in labour to prevent sepsis among pregnant women undergoing vaginal delivery in Nigeria (AZIN-V): a study protocol for a hybrid type 2 effectiveness-implementation trial
Source: BMJ Open. 2026 Feb 6;16(2):e110719. doi: 10.1136/bmjopen-2025-110719 (PMC12887512; doi:10.1136/bmjopen-2025-110719)
Supplement: online supplemental file 1 [file bmjopen-16-2-s001.docx]

## APPENDIX 1a: Informed Consent Document – Intervention Group

Title of the research: Azithromycin use in labour to prevent sepsis among pregnant women undergoing vaginal births in Nigeria (AZIN-V): a cluster-randomised hybrid type-2 effectiveness implementation trial.

Name(s) and affiliation(s) of researcher(s): Professor Bosede B. Afolabi, Chief Investigator, Dept. of Obstetrics and Gynaecology, College of Medicine of University of Lagos and Lagos University Teaching Hospital, Idi-Araba, Lagos.

Sponsor(s) of research: College of Medicine of the University of Lagos

Purpose(s) of research: The purpose of this study is to find out if taking an oral antibiotic called azithromycin during labour is better at preventing serious infections in mothers compared to usual care without azithromycin. The term “usual care” refers to healthcare services routinely provided to you at this hospital if you were not taking part in this study. We want to see if oral azithromycin is safe, effective, and affordable for pregnant women giving birth naturally in Nigeria. By joining this study, you will help us understand if azithromycin can help keep mothers, and their babies, healthier during and after childbirth.

Why we seek your participation to be part of the AZIN-V study: We are asking you to be part of this study because we want to learn how to better protect mothers from serious infections during and after childbirth. You will receive the usual care provided in this hospital plus oral azithromycin during labour and childbirth. You and other pregnant women in this hospital will be compared to other pregnant women who will receive usual care only. Your participation will help us understand if using oral azithromycin, an antibiotic, can improve the health and safety of mothers giving birth naturally. By joining this study, you will contribute to important research that could benefit many other women in Nigeria and around the world. Your involvement is crucial in helping us find better ways to keep mothers and their babies healthy and safe from infections during and in the period following childbirth.

Procedure of the research including what shall be required of each participant and approximate total number of participants that would be involved in the research: We would like to ask you some questions, the answers for which will be entered into an electronic database for the trial. We think this initial assessment will take approximately 15 minutes of your time. We will also retrieve additional relevant information from your case note or medical records, where necessary. As part of the intervention group, a single dose of 2g azithromycin will be given to you by a research nurse who will observe you take it by mouth with water provided from a sealed 50mL bottle. We will update your records each at each visit/encounter, from your antenatal clinic and/or labour ward records. We will monitor and check on you for any complaint that suggests you might have serious infection or sepsis. If the site investigator suspects sepsis (serious infection) and recommends it, we will take a sample of your blood which will be approximately 35mL, urine, pus, or other appropriate biological samples to check for the cause, site, and severity of the sepsis. We will also follow up whenever you are on admission to monitor your progress and update your records. We will follow you up until 6 weeks after you have delivered. After discharge from the hospital, we will contact you through phone or physical visit you at your home to get additional information related to maternal sepsis and maternal infection.

In total you will be required to make four (4) study-related clinic visits after your first contact with the research team or we will make home visits, at your convenience. If for any reason you miss a visit that was agreed to be done at the clinic, we will try to reach you or a relative (whom you suggested at your first encounter) by calling you on the phone to find out why. If we are unable to get you on the phone, we will visit you at home to make enquiries regarding your missed visit and your wellbeing and collect the necessary information and blood specimens during the visit. A total of 5,040 participants will be involved in this research.

Expected duration of research and of participant(s)’ involvement: In total, we expect you to be involved in this research for 6 weeks. You should not spend more than 1 hour at each clinic or home visit.

Risk(s): This study involves collecting your information in an electronic database designed for the study and if your doctor recommends it, we will collect some blood samples. Therefore, the harm it poses to participants is minimal. The trial drugs too have been found to be safe for use during labour and has been used in another research involving more than one country with no serious side effect documented so far. You are however free to call the investigators immediately (see contact details at the end of this pamphlet) if you notice any complaint/symptom, you are not sure of, or suspect may be a side effect of the medication. The product information leaflet for participants contains the list of potential side effects of the medication (azithromycin).

Costs to the participants, if any, of joining the research: Your participation in this research will not cost you anything as you will not be required to pay any fee for participating in the research, for investigations, or for drugs. For each study visit, you will be reimbursed the cost of transportation and time spent for the study participation.

Benefit(s):

**To the Participant:** As a participant in the AZIN-V study, you will receive free blood tests and study medications. You will benefit from personalized attention through one-on-one contact with healthcare providers, with phone numbers exchanged for easy communication. Additionally, you will receive regular appointment reminders via phone calls or text messages to help you keep track of clinic visits. Overall, your health will be closely monitored throughout the study, ensuring you receive comprehensive care.

**To the Larger Research Community:** The results of this research could lead to changes in current practices for preventing maternal sepsis during and after delivery, improving the effectiveness of azithromycin use during labour, and potentially lowering the high maternal death rate in our environment. The findings will be shared at conferences to enhance scientific knowledge among healthcare workers and published in reputable medical journals for broader dissemination, contributing to the global understanding of maternal health and infection prevention.

Confidentiality: All information obtained in this study will be kept strictly confidential by the principal investigator. Data will be stored in pass-worded electronic database. Your personal details will not be released in any publication or reports arising from this study.

Voluntariness: Your participation in this research is entirely voluntary. You have a right to agree or decline to participate. Your refusal to participate will not in any way affect the quality of care you receive at the hospital.

Alternatives to participation: There are no alternative options for participating in this research. You can either choose to participate in the study or decide not to participate.

Due inducement(s): You will not be paid any fees for participating in this research.

Consequences of participants’ decision to withdraw from research and procedure for orderly Termination of participation: You may choose to withdraw from the study at any time. Please note that some of the information that has been obtained about you before you chose to withdraw will be used in reports and publications. However, the researchers promise to make good effort to comply with your wishes as much as is practicable. You only need to notify the research team at your hospital of your decision to withdraw if need be. It is however highly recommended you complete the research unless you have a strong reason to do otherwise.

Modality of providing treatments and action(s) to be taken in case of injury or adverse event(s):

If you suffer any injury because of your participation in this research, for instance a severe side effect confirmed to be due to the study drug, you will be treated at the hospital where you were enrolled for the study or referred to another hospital involved in the research if necessary, and the researchers will bear the cost of such treatment.

What happens to research participants and communities when the research is over: The researcher will inform you of the outcome of the research. Important findings from the research may be used in building up health talks for pregnant women receiving antenatal care at various health facilities in Nigeria.

Statement about sharing of benefits among researchers and whether this includes or exclude research participants: There is no benefit either in cash or kind to be shared among the researchers or among the participants as a motivating factor or inducement to partake in this research. The researchers also hereby declare that they are not deriving any benefit from the drug manufacturers for the use of specific drugs in the conduct of this research.

Any apparent or potential conflict of interest: The researchers have no competing interest in conducting this research.

Central storage and future use of blood samples and study information: If a small portion of the blood sample is collected from you, it will be stored for a long time at the College of Medicine, University of Lagos Biospecimen Repository for current and future research that might include genetic research in which case your individual genetic results or incidental findings will not be shared with you. These tests will be research-related, and the findings may need additional research before their clinical significance is understood, and appropriate actions are determined. Data from the blood analysis from archived samples will be stored without identifiers and may be shared with secondary researchers within or outside the study sites.

Study data & document retention: A copy of the signed original informed consent documents for each participant, REDCap data and original copies of other study documentation (e.g., drug inventory forms, participant clinic records, laboratory reports, etc.) will be retained by the Chief Investigator for a minimum of 10 years.

For further enquiry, please contact:

Researcher’s Contact:

Chief Investigator:

Prof. Bosede B. Afolabi

Dept. of Obstetrics and Gynaecology,

College of Medicine, University of Lagos/

Lagos University Teaching Hospital,

Idi-Araba, Lagos

Mobile Contact: +2348076960670

E-mail: [bbafolabi@unilag.edu.ng](mailto:bbafolabi@unilag.edu.ng)

Clinical Governance Lead

Dr Christian C. Makwe

Dept. of Obstetrics and Gynecology,

College of Medicine, University of Lagos/

Lagos University Teaching Hospital,

Idi-Araba, Lagos.

Mobile Contact: +2348033358021

E-mail: ccmakwe@unilag.edu.ng

xxxxxx State Principal Investigator:

Dr. xxxxx xxxxxxxx

Dept. of Obstetrics and Gynaecology

xxxxxxx University Teaching Hospital,

Mobile Contact: +234XXXXXXXXXX

E-mail:

LUTH Health Research Ethics Committee’s Contact:

Room 107, Administrative Block, Lagos University Teaching Hospital

Idi-Araba, Lagos

Informed consent form

PARTICIPANT’S STUDY ID: __________________

STATEMENT OF PERSON (RESEARCH STAFF) OBTAINING INFORMED CONSENT:

I have fully explained this research to ____________________________________ and have given sufficient information, including information about risks and benefits, to make an informed decision.

DATE: __________________ SIGNATURE: _______________________________

NAME: _____________________________________________________________

STATEMENT OF PERSON (PARTICIPANT) GIVING CONSENT:

I have read the description of the research or have had it read and translated to me in a language I understand. I have also talked it over with the study staff to my satisfaction. I understand that my participation is voluntary. I know enough about the purpose, methods, risks, and benefits of the research study to judge that I want to take part in it. I understand that I may freely stop being part of this study at any time.

Consent for storage of samples for future research (tick the option of your choice):

I DO NOT want my blood sample to be stored and with my data shared with other scientists.
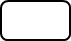


My blood sample can be stored and with my de-identified data can be shared with other scientists for future research related only to the current research project.
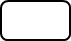


My blood sample can be stored and with my de-identified data shared with other scientists for future research of any type which has been properly approved.
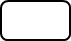


I was given a copy of this consent form and the information sheet to keep for myself.
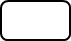


DATE: ________________ SIGNATURE: _________________________________

NAME: _____________________________________________________________

Thumbprint (if applicable):

WITNESS’ NAME (if applicable): ________________________________________

WITNESS’ SIGNATURE & DATE (if applicable): ___________________________

## APPENDIX 1b: Informed Consent Document – Control Group

Title of the research: Azithromycin use in labour to prevent sepsis among pregnant women undergoing vaginal births in Nigeria (AZIN-V): a cluster-randomised hybrid type-2 effectiveness implementation trial.

Name(s) and affiliation(s) of researcher(s): Professor Bosede B. Afolabi, Chief Investigator, Dept. of Obstetrics and Gynaecology, College of Medicine of University of Lagos and Lagos University Teaching Hospital, Idi-Araba, Lagos.

Sponsor(s) of research: College of Medicine of the University of Lagos

Purpose(s) of research: The purpose of this study is to find out if taking an oral antibiotic called azithromycin during labour is better at preventing serious infections in mothers compared to usual care. The term “usual care” refers to healthcare services routinely provided to you at this hospital if you were not taking part in this study. We are conducting this study to see if the use oral azithromycin is safe, effective, and affordable for pregnant women giving birth naturally in Nigeria. Sometimes microorganisms called bacteria can cause serious illness or infection in mothers and their babies during and after childbirth. By joining this study, you will help us understand if azithromycin can help keep mothers, and their babies, healthier during and after childbirth.

Why we seek your participation to be part of the AZIN-V study: We are asking you to be part of this study because we want to learn how to better protect mothers from serious infections during and after childbirth. You will receive the usual care provided in this hospital and azithromycin will not be given to you during labour and childbirth. You and other pregnant women in this hospital will be compared to other pregnant women who will be given azithromycin. Your participation will help us understand if using oral azithromycin, an antibiotic, can improve the health and safety of mothers giving birth naturally. By joining this study, you will contribute to important research that could benefit many other women in Nigeria and around the world. Your involvement is crucial in helping us find better ways to keep mothers and their babies healthy and safe from infections during and in the period following childbirth.

Procedure of the research including what shall be required of each participant and approximate total number of participants that would be involved in the research: We would like to ask you some questions, the answers for which will be entered into an electronic database for the trial. We think this initial assessment will take approximately 15 minutes of your time. We will also retrieve additional relevant information from your case note or medical records, where necessary. As part of the usual care group, you will receive the standard care provided by skilled healthcare practitioners to prevent infections during labour. This care includes the usual infection prevention and control practices routinely provided in this health facility. We will update your records each at each visit/encounter, from your antenatal clinic and/or labour ward records. We will monitor and check on you for any complaint that suggests you might have serious infection or sepsis. If the site investigator suspects sepsis (serious infection) and recommends it, we will take sample of your blood which will be approximately 35mls, urine, pus, or other appropriate biological samples to check for the cause, site, and severity of the sepsis. We will also follow up whenever you are on admission to monitor your progress and update your records. We will follow you up until 6 weeks after you have delivered. After discharge from the hospital, we will contact you through phone or physical visit you at your home to get additional information related to maternal sepsis and maternal infection.

In total you will be required to make four (4) study-related clinic visits after your first contact with the research team or we will make home visits, at your convenience. If for any reason you miss a visit that was agreed to be done at the clinic, we will try to reach you or a relative (whom you suggested at your first encounter) by calling you on the phone to find out why. If we are unable to get you on the phone, we will visit you at home to make enquiries regarding your missed visit and your wellbeing and collect the necessary information and blood specimens during the visit. A total of 5,040 participants will be involved in this research.

Expected duration of research and of participant(s)’ involvement: In total, we expect you to be involved in this research for 6 weeks. You should not spend more than 1 hour at each clinic or home visit.

Risk(s): This study involves collecting your information in an electronic database designed for the study and if your doctor recommends it, we will collect some blood samples. Therefore, the harm it poses to participants is minimal. The trial drugs too have been found to be safe for use during labour and has been used in another research involving more than one country with no serious side effect documented so far. You are however free to call the investigators immediately (see contact details at the end of this pamphlet) if you notice any complaint/symptom, you are not sure of, or suspect may be a side effect of the medication. The product information leaflet for participants contains the list of potential side effects of the medication (azithromycin).

Costs to the participants, if any, of joining the research: Your participation in this research will not cost you anything as you will not be required to pay any fee for participating in the research, for investigations, or for drugs. For each study visit, you will be reimbursed the cost of transportation and time spent for the study participation.

Benefit(s):

**To the Participant:** As a participant in the AZIN-V study, you will receive free blood tests and study medications. You will benefit from personalized attention through one-on-one contact with healthcare providers, with phone numbers exchanged for easy communication. Additionally, you will receive regular appointment reminders via phone calls or text messages to help you keep track of clinic visits. Overall, your health will be closely monitored throughout the study, ensuring you receive comprehensive care.

**To the Larger Research Community:** The results of this research could lead to changes in current practices for preventing maternal sepsis during and after delivery, improving the effectiveness of azithromycin use during labour, and potentially lowering the high maternal death rate in our environment. The findings will be shared at conferences to enhance scientific knowledge among healthcare workers and published in reputable medical journals for broader dissemination, contributing to the global understanding of maternal health and infection prevention.

Confidentiality: All information obtained in this study will be kept strictly confidential by the principal investigator. Data will be stored in pass-worded electronic database. Your personal details will not be released in any publication or reports arising from this study.

Voluntariness: Your participation in this research is entirely voluntary. You have a right to agree or decline to participate. Your refusal to participate will not in any way affect the quality of care you receive at the hospital.

Alternatives to participation: There are no alternative options for participating in this research. You can either choose to participate in the study or decide not to participate.

Due inducement(s): You will not be paid any fees for participating in this research.

Consequences of participants’ decision to withdraw from research and procedure for orderly

Termination of participation: You may choose to withdraw from the study at any time. Please note that some of the information that has been obtained about you before you chose to withdraw will be used in reports and publications. However, the researchers promise to make good effort to comply with your wishes as much as is practicable. You only need to notify the research team at your hospital of your decision to withdraw if need be. It is however highly recommended you complete the research unless you have a strong reason to do otherwise.

Modality of providing treatments and action(s) to be taken in case of injury or adverse event(s):

If you suffer any injury because of your participation in this research, for instance a severe side effect confirmed to be due to the study drug, you will be treated at the hospital where you were enrolled for the study or referred to another hospital involved in the research if necessary, and the researchers will bear the cost of such treatment.

What happens to research participants and communities when the research is over: The researcher will inform you of the outcome of the research. Important findings from the research may be used in building up health talks for pregnant women receiving antenatal care at various health facilities in Nigeria.

Statement about sharing of benefits among researchers and whether this includes or exclude research participants: There is no benefit either in cash or kind to be shared among the researchers or among the participants as a motivating factor or inducement to partake in this research. The researchers also hereby declare that they are not deriving any benefit from the drug manufacturers for the use of specific drugs in the conduct of this research.

Any apparent or potential conflict of interest: The researchers have no competing interest in conducting this research.

Central storage and future use of blood samples and study information: If a small portion of the blood sample is collected from you, it will be stored for a long time at the College of Medicine, University of Lagos Biospecimen Repository for current and future research that might include genetic research in which case your individual genetic results or incidental findings will not be shared with you. These tests will be research-related, and the findings may need additional research before their clinical significance is understood, and appropriate actions are determined. Data from the blood analysis from archived samples will be stored without identifiers and may be shared with secondary researchers within or outside the study sites.

Study data & document retention: A copy of the signed original informed consent documents for each participant, REDCap data and original copies of other study documentation (e.g., drug inventory forms, participant clinic records, laboratory reports, etc.) will be retained by the Chief Investigator for a minimum of 10 years.

For further enquiry, please contact:

Researcher’s Contact:

Chief Investigator:

Prof. Bosede B. Afolabi

Dept. of Obstetrics and Gynaecology,

College of Medicine, University of Lagos/

Lagos University Teaching Hospital,

Idi-Araba, Lagos

Mobile Contact: +2348076960670

E-mail: [bbafolabi@unilag.edu.ng](mailto:bbafolabi@unilag.edu.ng)

Clinical Governance Lead

Dr Christian C. Makwe

Dept. of Obstetrics and Gynecology,

College of Medicine, University of Lagos/

Lagos University Teaching Hospital,

Idi-Araba, Lagos.

Mobile Contact: +2348033358021

E-mail: ccmakwe@unilag.edu.ng

xxxxxx State Principal Investigator:

Dr. xxxxx xxxxxxxx

Dept. of Obstetrics and Gynaecology

xxxxxxx University Teaching Hospital,

Mobile Contact: +234XXXXXXXXXX

E-mail:

LUTH Health Research Ethics Committee’s Contact:

Room 107, Administrative Block, Lagos University Teaching Hospital

Idi-Araba, Lagos

Informed consent form

PARTICIPANT’S STUDY ID: __________________

STATEMENT OF PERSON (RESEARCH STAFF) OBTAINING INFORMED CONSENT:

I have fully explained this research to ____________________________________ and have given sufficient information, including information about risks and benefits, to make an informed decision.

DATE: __________________ SIGNATURE: _______________________________

NAME: _____________________________________________________________

STATEMENT OF PERSON (PARTICIPANT) GIVING CONSENT:

I have read the description of the research or have had it read and translated to me in a language I understand. I have also talked it over with the study staff to my satisfaction. I understand that my participation is voluntary. I know enough about the purpose, methods, risks, and benefits of the research study to judge that I want to take part in it. I understand that I may freely stop being part of this study at any time.

Consent for storage of samples for future research (tick the option of your choice):

I DO NOT want my blood sample to be stored and with my data shared with other scientists.
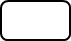


My blood sample can be stored and with my de-identified data can be shared with other scientists for future research related only to the current research project.
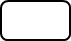


My blood sample can be stored and with my de-identified data shared with other scientists for future research of any type which has been properly approved.
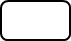


I was given a copy of this consent form and the information sheet to keep for myself.
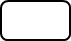


DATE: ________________ SIGNATURE: _________________________________

NAME: _____________________________________________________________

Thumbprint (if applicable):

WITNESS’ NAME (if applicable): ________________________________________

WITNESS’ SIGNATURE & DATE (if applicable): ___________________________
