# Supplementary material for: Azithromycin use in labour to prevent sepsis among pregnant women undergoing vaginal delivery in Nigeria (AZIN-V): a study protocol for a hybrid type 2 effectiveness-implementation trial
Source: BMJ Open. 2026 Feb 6;16(2):e110719. doi: 10.1136/bmjopen-2025-110719 (PMC12887512; doi:10.1136/bmjopen-2025-110719)
Supplement: Supplementary file 2 [file bmjopen-2025-110719.reviewer_comments.pdf]

PEER REVIEW HISTORY

BMJ Open publishes all reviews undertaken for accepted manuscripts. Reviewers are asked to complete a checklist review form and are provided with free text boxes to elaborate on their assessment. These free text comments are reproduced below.

ARTICLE DETAILS

Title (Provisional)

Azithromycin use in labour to prevent sepsis among pregnant women undergoing vaginal delivery in Nigeria (AZIN-V): study protocol for a hybrid type-2 effectiveness–implementation trial

Authors

Afolabi, Bosede Bukola; MAKWE, CHRISTIAN CHIGOZIE Chigozie; Oluwole, Esther Oluwakemi; Obi-Jeff, Chisom; Mitchell, Eleanor J; Banke-Thomas, Aduragbemi; Adeyemo, Titilope Adenike; Abioye, Ajibola Ibraheem; Eboreime, Ejemai Amaize; Saidu, Abdulhadi Diyo; Okoro , Udo Abali; Akintan, Patricia; Osuagwu, Chioma Stella; Chieme, Chisom Florence; Lawanson , Teniola; Hossain, Anower; Walker, Kate

VERSION 1 - REVIEW

**Reviewer**	**1**
**Name**	**Phipps, Hala**
**Affiliation**	**Sydney Local Health District, Women and babies**
**Date**	**19-Nov-2025**
**COI**	**None**

bmjopen-2025-110719 _ **Reviewer Hala Phipps**

Thank you for giving me the opportunity to review this manuscript, "Study protocol for a hybrid type-2 effectiveness implementation trial: azithromycin use in labour to prevent sepsis among pregnant women undergoing vaginal delivery in Nigeria (AZIN-V)

The manuscript is very well-written; however, I have some queries and suggestions for the authors to consider:

Title: In my opinion the title needs to include *cluster-randomised* before the word trial. A potential title that provides clarity could be *"A hybrid type-2 effectiveness implementation cluster-randomised trial: azithromycin use in labour to prevent sepsis among pregnant women undergoing vaginal delivery in Nigeria (AZIN-V)- A study protocol.*

Feasibility of this large multi-centre trial is a major concern. I recognise that there was convenience sampling of clinician (10 Drs) and participants (20 pregnant women) that provided views on the study protocol, but most clinicians being the midwives/nurses that

provide most of the care were not consulted. No **pilot/feasibility study** has been conducted to assess recruitment capability or acceptability among clinicians and women—both critical to trial success.
Undertaking a **pilot intervention RCT** in an intrapartum setting, is a crucial step in preparing for a full-scale randomized controlled trial. The key reasons are it assesses feasibility and retention, intervention delivery and data collection. It also identifies logistical processes such as randomization, blinding and follow up schedules. In the Australian context, the **NHMRC typically does not fund** intrapartum RCTs that have not first demonstrated feasibility through a pilot

Re Data Safety Monitoring Committee (DSMC): It is likely the investigators have already set up the members but did not think to include the details here (3-5 individuals that include at least 1 clinician and a statistician. Members are chosen because they are experienced in trials and/or maternity research and are approved by the TSC).

In my opinion it is essential that a published protocol of such a large RCT should include a DSMC Charter as a supplementary file. This would provide a clear, transparent framework that ensures the scientific integrity of the study. y defining the committee's responsibilities, processes, and communication pathways, the charter helps prevent bias, ensures consistent handling of emerging issues, and fulfils ethical and regulatory requirements for high-risk or large-scale research.

Consenting of participants: As this is a cluster RCT, I fail to understand why a labouring women (fully dilated but not pushing) should be asked to sign a consent form. unless a woman has effective analgesia, she is in too much pain to consent. In addition, clinicians working in birthing suites and delivery wards operate under intense time pressures, with very limited capacity to support research activities. I highly recommend that the study research nurses/officers based at each hospital sites obtain written consent either antenatally or after a woman has given birth.

Implementation non-randomised trial: I commend the investigators as this is very important to undertake. I just have 1 query_ how were the 12 hospital sites selected? This information should be clearly outlined.

VERSION 1 - AUTHOR RESPONSE

Reviewers' comments	Authors' response	Previous versions	Page number
Title: In my opinion the title needs to include cluster-randomised before the word trial. A potential title that provides clarity could be "A hybrid type-2 effectiveness implementation cluster-randomised trial: azithromycin use in labour to prevent sepsis among pregnant women undergoing vaginal delivery in Nigeria (AZIN-V)- A study protocol. [NOTE FROM THE EDITORS: Feel free to rebut this suggestion, given that the cluster-randomisation applies only to the clinical trial and not the implementation study component. Alternatively, the authors can consider if it is possible to further rephrase the title to include this information accurately, keeping in mind the editorial request above concerning the title format]	"Azithromycin use in labour to prevent sepsis among pregnant women undergoing vaginal delivery in Nigeria (AZIN-V): study protocol for a hybrid type-2 effectiveness-implementation trial."<br><br>We changed the title to the above as recommended by a previous assessor but left out cluster randomised as it applies only to the clinical trial and not the implementation science aspect.	"Study protocol for a hybrid type-2 effectiveness-implementation trial: azithromycin use in labour to prevent sepsis among pregnant women undergoing vaginal delivery in Nigeria (AZIN-V)"	Page 2, 'Title'
Feasibility of this large multi-centre trial is a major concern. I recognise that there was convenience	Before the trial commenced, IDIs were conducted at two health facilities, involving purposively sampled **ten healthcare personnel (five nurses, four doctors, and one laboratory technician) and twenty** pregnant women, to understand their	Before the trial commenced, IDIs were conducted at two health facilities, involving purposively sampled doctors (n=10) and	Page 14, 'Patient and public involvement'

sampling of clinician (10 Drs) and participants (20 pregnant women) that provided views on the study protocol, but most clinicians being the midwives/nurses that provide most of the care were not consulted. No pilot/feasibility study has been conducted to assess recruitment capability or acceptability among clinicians and women—both critical to trial success.	views, expectations, concerns, and suggestions regarding the AZIN-V trial.<br><br>Although a pilot or feasibility was not conducted, a baseline prospective cohort study was performed prior to the AZIN-V Trial to determine the incidence and risk factors for maternal sepsis (manuscript in preparation).<br>Additionally, similar multi-centre trials involving pregnant and postpartum women have been successfully completed, achieving a retention rate exceeding 97% over six months of follow-up.<br>1. Afolabi BB, Babah OA, Adeyemo TA, et al. Intravenous versus oral iron for anaemia among pregnant women in Nigeria (IVON): an open-label, randomised controlled trial. Lancet Glob Health. 2024 Oct;12(10):e1649-e1659. doi: 10.1016/S2214-109X(24)00239-0.<br>2. Afolabi BB, Adaramoye VO, Adeyemo TA, et al. Intravenous ferric carboxymaltose versus oral ferrous sulphate for the treatment of moderate to severe postpartum anaemia in Nigerian women (IVON-PP): protocol for an open-label randomised controlled type 1 hybrid effectiveness-implementation trial. BMJ Open. 2024 Aug 17;14(8):e086553. doi: 10.1136/bmjopen-2024-086553.<br>3. Afolabi BB, Adaramoye VO, Adeyemo TA et al. Randomized trial of ferric carboxymaltose for postpartum women with moderate to severe anemia (IVON-PP) [version 1]. VeriXiv 2025, 2:169 (https://doi.org/10.12688/verixiv.1314.1)	pregnant women (n=20), to understand their views, expectations, concerns, and suggestions regarding the AZIN-V trial. The trial protocol was adapted based on their feedback.	
Undertaking a pilot intervention	AZIN-V Trial is a cluster trial design, with randomisation		Page 5, 'Study design',

RCT in an intrapartum setting, is a crucial step in preparing for a full-scale randomized controlled trial. The key reasons are it assesses feasibility and retention, intervention delivery and data collection. It also identifies logistical processes such as randomization, blinding and follow up schedules. In the Australian context, the NHMRC typically does not fund intrapartum RCTs that have not first demonstrated feasibility through a pilot.	conducted at the health facility level rather than for individual participants. Due to the nature of the intervention and study design, neither sites nor participants were blinded to allocation.<br>The research team has demonstrated effective participant follow-up and high retention rates in previous large multi-centre trials, such as the IVON Trial and IVON-PP Trial, as shown in the references above.		Page 9, 'Recruitment', Randomisation', 'Blinding', 'Data collection', Page 12, 'Study sites', 'Blinding'.
Re Data Safety Monitoring Committee (DSMC): It is likely the investigators have already set up the members but did not think to include the details here (3-5 individuals that include at least 1 clinician and a statistician. Members are chosen because they are experienced in trials and/or maternity research	The DSMB comprises of three members:<br>1. Christopher Robert Sudfeld (Chair)<br>Associate Professor of Global Health and Nutrition<br>Dept. of Global Health and Population Harvard T.H. Chan School of Public Health<br>2. Martin Meremikwu (Member)<br>Professor of Paediatrics and Child Health<br>College of Medical Sciences University of Calabar, Nigeria<br>3. Joanna Sturgess (Member)<br>Research Fellow in the Medical Statistics Department at London School of Hygiene and Tropical Medicine.		

and are approved by the TSC).	The TSC comprises of three members:<br>1. Paul Heath (Chair)<br>Professor of Paediatric Infectious Diseases,<br>St. George's University of London<br>2. James Dodd (Member)<br>Medical Statistician<br>Liverpool School of Tropical Medicine<br>3. Nabila Youssouf (Member)<br>Research Associate<br>London School of Hygiene and Tropical Medicine<br>4. Oliver Ezechi (Member)<br>Professor and Director of Research<br>Nigerian Institute of Medical Research, Lagos, Nigeria.<br>5. Zahida Qureshi (Member)<br>Associate Professor of Obstetrics and Gynaecology,<br>Faculty of Health Sciences<br>University of Nairobi, Kenya.		
In my opinion it is essential that a published protocol of such a large RCT should include a DSMC Charter as a supplementary file. This would provide a clear, transparent framework that ensures the scientific integrity of the study. y defining the committee's responsibilities, processes, and communication pathways, the charter helps prevent bias,	The DSMB charter will be provided as a supplementary file and made available via the Open Science Framework (OSF) repository.		

ensures consistent handling of emerging issues, and fulfils ethical and regulatory requirements for high-risk or large-scale research.			
Consenting of participants: As this is a cluster RCT, I fail to understand why a labouring women (fully dilated but not pushing) should be asked to sign a consent form. Unless a woman has effective analgesia, she is in too much pain to consent. In addition, clinicians working in birthing suites and delivery wards operate under intense time pressures, with very limited capacity to support research activities. I highly recommend that the study research nurses/officers based at each hospital sites obtain written consent either antenatally or after a woman has given birth.	Potential participants will receive counselling and study information during routine antenatal visits. Upon admission in active labour, an information video will be shown to reinforce this process. Informed written consent will be obtained intrapartum, specifically during the active phase of the first stage and the non-pushing phase of the second stage of labour. The non-pushing phase may last up to an hour, during which labour characteristics are comparable to the active phase of the first stage. Women in the pushing phase will not be eligible to provide consent for trial participation.<br>This study, including the consent process, has received approval from independent health research ethics committees (the National Health Research Ethics Committee, Nigeria, the ethics committees of the participating health institutions), and the States's health ethics board. Additionally, regulatory approval was obtained from the National Agency for Food and Drug Administration and Control in Nigeria.<br><br>*Ethical considerations*<br>Ethical approval has been obtained from the National Health Research Ethics Committee, Nigeria (NHREC/01/01/2007 - 30/09/2024), the ethics committees of the **participating** health institutions **[Lagos University Teaching Hospital Research Ethics Committee: ADM/DSCST/HREC/APP/6325;**	Recruitment<br>All pregnant women are approached during routine antenatal care visits and labour by trained research staff who will provide study information and discuss participation. A short information video will also be shown to potential participants.<br>Pregnant women admitted in the active phase of labour at the study sites are screened for eligibility to participate in the trial, with consent obtained intrapartum before the onset of the pushing phase of labour. The informed consent form was translated into Hausa, Igbo, Yoruba, and Pidgin English, the most popular languages used in the participating facilities.<br><br>*Ethical considerations*<br>Ethical approval has been obtained from	Page 2 & 3, 'Ethics and dissemination', Page 9, 'Recruitment', Page 14 & 15, 'Ethical considerations'.

	**University of Ilorin Teaching Health Research Ethics Committee: ERC/PAN/2025/03/0581; University of Benin Teaching Hospital Health Research Ethics Committee: ADM/E22/A/VOL. VII/483117141; Aminu Kano Teaching Hospital, Research Ethics Committee: AKTH/MAC/SUB/12A/P-3/VI/2509, Aminu Kano Teaching Hospital, Research Ethics Committee: AKTH/MAC/SUB/12A/P-3/VI/2509; Irrua Specialist Teaching Hospital; ISTH/HREC/20241507/605]**, and the States' health ethics boards. Regulatory approval was obtained from the National Agency for Food and Drug Administration and Control. The trial is duly registered in the International Standard Randomised Controlled Trial Number (ISRCTN16415327) registry.<br>Written consent for participation, including consent for community tracking and assessing health records, is obtained from all eligible women.[64 65] Women aged between 15 and 18 years are considered capable of providing consent, as they are considered emancipated for the purposes of this study, according to the guidelines of the NHREC and the 2016 WHO International Ethical Guidelines for Health-Related Research Involving Humans. Additional consent for de-identified data and samples is obtained for future studies or data sharing with secondary researchers.	the National Health Research Ethics Committee, Nigeria (NHREC/01/01/2007 - 30/09/2024), the ethics committees of the health institutions, and the States' health ethics boards. Regulatory approval was obtained from the National Agency for Food and Drug Administration and Control. The trial is duly registered in the International Standard Randomised Controlled Trial Number (ISRCTN16415327) registry.<br>Written consent for participation, including consent for community tracking and assessing health records, is obtained from all eligible women.[64 65] Women aged between 15 and 18 years are considered capable of providing consent, as they are considered emancipated for the purposes of this study, according to the guidelines of the NHREC and the 2016 WHO International Ethical Guidelines for Health-Related Research Involving Humans. Additional consent for de-	

		identified data and samples is obtained for future studies or data sharing with secondary researchers.	
Implementation non-randomised trial: I commend the investigators as this is very important to undertake. I just have 1 query: how were the 12 hospital sites selected? This information should be clearly outlined.	The 12 sites were purposively selected within the six states **from a pool of facilities where a pre-site readiness assessment was conducted** to account for health systems-based organisation factors. The inclusion criteria were 1) facility level (primary, secondary, and tertiary) and facility ownership: public (government-owned) and private (individual/charity/faith-based/non-government organisation-owned), 2) provision of maternity services (antenatal care, childbirth, and postnatal care services), 3) mixture of high, medium, and low number of vaginal births, and 4) location (rural, semi-urban, and urban).	Our trial is implemented in the 12 purposively selected sites within the six states to account for health systems-based organisation factors. The inclusion criteria were 1) facility level (primary, secondary, and tertiary) and facility ownership: public (government-owned) and private (individual/charity/faith-based/non-government organisation-owned), 2) provision of maternity services (antenatal care, childbirth, and postnatal care services), 3) mixture of high, medium, and low number of vaginal births, and 4) location (rural, semi-urban, and urban).	Page 12, 'Patient and public involvement'
COI statement(s):<br><br>Reviewer: 1<br>If you have selected 'Yes' above, please provide details of any competing interests.: N/A.	COI: None<br>Detail of any competing interests: N/A		Page 21

VERSION 2 - REVIEW

**Reviewer**	**1**
**Name**	**Phipps, Hala**
**Affiliation**	**Sydney Local Health District, Women and babies**
**Date**	**05-Dec-2025**
**COI**	

Thanks for addressing all the comments/ Queries.

I am rather surprised the ethics committee in Nigeria approves a woman in labour being consented for research. No ethics committee in Australia would ever allow this consenting strategy.

Re DSMB- I assume a brief paregraph of the members will be added to the protocol

VERSION 2 - AUTHOR RESPONSE

**Reviewers' comments**	**Authors' response**	**Previous versions**	**Page number, Section**
Thanks for addressing all the comments/queries.	We greatly appreciate your review and comments. Thank you.		
I am rather surprised the ethics committee in Nigeria approves a woman in labour being consented for research. No ethics committee in Australia would ever allow this consenting strategy.	We understand the reviewers' perspectives and during the trial development phase, we explored alternative approaches to consent.<br>The AZIN-V protocol was approved by several independent human research ethics committees and the trained research staff further ensure that the consent process during labour is conducted in a sensitive and respectful manner.<br>We also noted that similar intrapartum consent process/strategy has been deployed in a previous study: "Tita ATN, Carlo WA, McClure EM, Mwenechanya M, Chomba E, Hemingway-Foday JJ, Kavi A, et al. Azithromycin to Prevent Sepsis or Death in Women Planning a Vaginal Birth. N Engl J Med. 2023 Mar 30;388(13):1161-1170. doi: 10.1056/NEJMoa2212111."		
Re DSMB - I assume a brief paragraph of the members will be added to the protocol.	The Trial Management Group (TMG) are responsible for ensuring high-quality delivery of trial in accordance with the protocol and addressing problems promptly. The TMG includes the chief investigator, co-investigators, the trial manager and data manager. The TMG reports to the independent Trial Steering Committee (TSC) and the independent Data and Safety Monitoring Board (DSMB). The TSC will provide independent oversight and advice to the trial, while the DSMB is responsible for monitoring the trial data including safety and data integrity. The independent DSMB will monitor all SAEs and review SAE events by masked treatment group at each meeting and report to the TSC. **The DSMB will consist of a global health**	The Trial Management Group (TMG) are responsible for ensuring high-quality delivery of trial in accordance with the protocol and addressing problems promptly. The TMG includes the chief investigator, co-investigators, the trial manager and data manager. The TMG reports to the independent Trial Steering Committee (TSC) and the independent Data and	Page 14, Research governance

	**physician (Christopher Robert Sudfeld, Harvard T.H. Chan School of Public Health, USA), a paediatrician (Martin Meremikwu, University of Calabar, Nigeria), and a medical biostatistician (Joanna Sturgess, London School of Hygiene and Tropical Medicine, UK).** Auditing of research records will be done by the monitoring committees as outlined above, while financial auditing will be done by the Research Management Office and Audit Department of University of Lagos.	Safety Monitoring Board (DSMB). The TSC will provide independent oversight and advice to the trial, while the DSMB is responsible for monitoring the trial data including safety and data integrity. The independent DSMB will monitor all SAEs and review SAE events by masked treatment group at each meeting and report to the TSC. Auditing of research records will be done by the monitoring committees as outlined above, while financial auditing will be done by the Research Management Office and Audit Department of University of Lagos.	
